# Outcomes research resources in India: current status, need and way forward

**DOI:** 10.1186/2193-1801-2-518

**Published:** 2013-10-07

**Authors:** Jatin Shah, Akshay Pawaskar, Smit Kumar, Nilima Kshirsagar

**Affiliations:** Maharashtra University of Health Sciences, Nashik, Maharashtra India; Kalpavriksha Healthcare And Research, Thane, India; VIS Research Pvt Ltd, Thane, India; Clinical Pharmacology, Indian Council of Medical Research, Government of India, New Dehli, India; ESI-PGIMSR MGM Hospital, Government of India, Mumbai, India

**Keywords:** Outcomes research, Research resources, India

## Abstract

**Background:**

Despite their importance, the number of outcomes research studies conducted in India are lesser than other countries. Information about the distribution of existing outcomes research resources and relevant expertise can benefit researchers and research groups interested in conducting outcomes research studies and policy makers interested in funding outcomes research studies in India. We have reviewed the literature to identify and map resources described in outcomes research studies conducted in India.

**Methods:**

We reviewed the following online biomedical databases: Pubmed, SCIRUS, CINAHL, and Google scholar and selected articles that met the following criteria: published in English, conducted on Indian population, providing information about outcomes research resources (databases/registries/electronic medical records/electronic healthcare records/hospital information systems) in India and articles describing outcomes research studies or epidemiological studies based on outcomes research resources. After shortlisting articles, we abstracted data into three datasets viz. 1. Resource dataset, 2. Bibliometric dataset and 3. Researcher dataset and carried out descriptive analysis.

**Results:**

Of the 126 articles retrieved, 119 articles were selected for inclusion in the study. The tally increased to 133 articles after a secondary search. Based on the information available in the articles, we identified a total of 91 unique research resources. We observed that most of the resources were Registries (62/91) and Databases ( 23/91) and were primarily located in Maharashtra (19/91) followed by Tamil Nadu (11/91), Chandigarh (8/91) and Kerala (7/91) States. These resources primarily collected data on Cancer (44/91), Stroke (5/91) and Diabetes (4/91). Most of these resources were Institutional (38/91) and Regional resources (35/91) located in Government owned and managed Academic Institutes/Hospitals (57/91) or Privately owned and managed non – Academic Institutes/Hospitals (14/91). Data from the Population based Cancer Registry, Mumbai was used in 41 peer reviewed publications followed by Population based Cancer Registry, Chennai (17) and Rural Cancer Registry Barshi (14). Most of the articles were published in International journals (139/193) that had an impact factor of 0–1.99 (43/91) and received an average of 0–20 citations (55/91). We identified 193 researchers who are mainly located in Maharashtra (37/193) and Tamil Nadu (24/193) states and Southern (76/193) and Western zones (47/193). They were mainly affiliated to Government owned & managed Academic Institutes /Hospitals (96/193) or privately owned and managed Academic Institutes/ Hospitals (35/193).

**Conclusions:**

Given the importance of Outcomes research, relevant resources should be supported and encouraged which would help in the generation of important healthcare data that can guide health and research policy. Clarity about the distribution of outcomes research resources can facilitate future resource and funding allocation decisions for policy makers as well as help them measure research performance over time.

**Electronic supplementary material:**

The online version of this article (doi:10.1186/2193-1801-2-518) contains supplementary material, which is available to authorized users.

## Introduction

Outcomes research is concerned with determining the end results and in turn the effectiveness of healthcare practices, interventions and systems. Outcomes research focuses on topics ranging from effectiveness, appropriateness, access, quality of care, quality of life, health status, disease prevention, screening, drug treatment, medical procedures, medical practices, diagnostic tests, guidelines and healthcare policy (Jefford et al. 2003).

Despite the role of outcomes research studies in discerning practice variation (Pilote and Tager 2002), quality of care and determining “What actually works”, a quick literature search in Gopubmed (GoPubMed® 2013) conducted by the authors revealed that the number of outcomes research studies in India have been lower in comparison to countries like USA, UK and Germany. (9362, 195663, 46028, 36693 respectively). (Search strategy: “Outcome Assessment (Health Care)”[mesh], Gopubmed,) Although there is a lack of studies evaluating the reason behind this trend, paucity of funding, non availability of infrastructure, lack of relevant expertise and trained staff could be factors responsible for this trend. Even when these factors are available, there exists a significant disconnect amongst them as data about existing resources is not widely accessible. If made available, information about existing resources can help 1. Policy makers to plan efficient strategies that can build up on existing outcomes research resources thus ensuring economies of scale as well as predict resource use and improve efficiency in the allocation of resources (Liu et al. 2008). 2. Researchers and research groups become aware of existing research resources thus avoiding duplication and facilitating higher productivity at a lower cost. Despite the importance of this information, till date no previous study has worked on compiling and sharing this information in a systematic manner.

India currently faces a mixed burden of both communicable and non-communicable diseases, the latter responsible for two-third of the total morbidity burden and more than half (53%) of total mortality in India. (WHO 2013) This dual burden poses significant public health challenges before India like safeguarding public health, expanding health care coverage and improving quality of care while controlling costs. Given the current economic downturn, Indian policy makers need to take cue from Australia, Japan, South Korea and China where Outcomes Research data are used for setting national policy, designing drug formulary and drafting pharmaceutical economics guidelines. (Garman 2013) In order to encourage and facilitate the conduct of outcomes research studies, knowledge, access and sustained support of pre-existing resources is essential. Few studies have mapped outcomes research resources in India.

In order to bridge this gap, we carried out a review of the literature to identify and map resources described in previous outcomes research studies conducted in India.

## Methods

### Ethics

We carried out a review of published literature and hence did not seek ethics approval for this review.

### Definitions

Outcomes research resources include outcomes researchers, infrastructure, trained staff/manpower and electronic data sources like databases, registries, electronic health records, electronic medical records and hospital information systems. Data on researcher and electronic resources are reported in published literature but data on infrastructure and manpower is rarely published. Accordingly, we decided to focus on the former. For the purpose of this study, we used the following definitions of outcomes research resources:

Databases and Registries that collect data as a part of clinical practice or for research purposes. We used the following operational definition for a biomedical registry: A system for the registration, record keeping and referral of biomedical data, material or resources. (Dict.md, Medical dictionary, 2013)
(i) Electronic medical records defined as computerized systems that collect, manage and deliver healthcare data and information in electronic format as a part of routine practice (Luo 2006) (Rustagi and Singh 2012) and (ii) Electronic Healthcare records defined as a “comprehensive, cross-institutional, longitudinal collection of a patient’s health and healthcare data”. (Hoerbst and Ammenwerth 2010)
Experts – Details about researchers who carried out outcomes research studies using prospective or retrospective study designs.

### Search strategy

Two reviewers (JS and AP) having previous experience in the conduct of reviews carried out an independent search in the following online biomedical databases: Pubmed, 1985 to 2012 (Home - PubMed - NCBI 2013), SCIRUS, 1980 to 2012 (Scirus search engine for scientific information 2013); CINAHL, 1985 to 2012 (CINAHL | Cumulative Index to Nursing and Allied Health | EBSCO 2013) and Google scholar (Google Scholar 2013). The cut-off dates for each database vary. They indicate the period of availability of articles in each of the databases.

We combined the following keywords and their MESH terms using Boolean operators to build a search strategy: Outcomes, Database, Registry, Electronic Medical records, Electronic Healthcare records, Hospital Information systems and India. Details of the search strategy are described below.

Search strategies:

- “Outcomes” AND (“Database” [Publication Type]) OR “Registries"[Mesh]) AND India
- outcomes AND database AND India
- outcomes AND registry AND India
- (registry OR database) AND India AND “outcomes research”
- (registry OR database) AND India AND “outcomes research”
- (electronic health records) AND India
- (electronic medical records) AND India
- (electronic healthcare records) AND india
- (India) AND electronic medical record[MeSH Terms]

### Eligibility criteria

We defined criteria that would help us filter through the initial list of search results and identify articles that would provide us the required data.

We used the following inclusion criteria:

1. Articles published in English language,
2. Articles reporting outcomes research studies,
3. Articles reporting studies conducted using Indian data,
4. Articles providing information about outcomes research or epidemiological resources (databases/registries/electronic medical records/electronic healthcare records/hospital information systems) in India,
5. Articles describing epidemiological studies based on outcomes research resources (databases/registries/electronic medical records/electronic healthcare records/hospital information systems).

In case when a full text version of the article was unavailable, we included the abstract if it provided detailed information about the study. Articles retrieved by applying the search strategy were screened first by title, then by abstract and later by reviewing their full text version. At each step, articles dissatisfying the selection criteria were excluded. The shortlisted articles retrieved by each reviewer were compared and disagreements were resolved by discussion and mutual consent. Based on data present in the shortlisted articles, we compiled a list of outcomes research resources (Additional file 1). In order to cross-check our search results, we searched for all outcomes research articles published using these resources. We applied the selection criteria to the results of this secondary search and included articles that our search strategy missed during the primary search.

### Data collection and data items

Two reviewers (JS and AP) independently reviewed each shortlisted article and captured information about the variables of interest in separate spreadsheets. The resulting data abstraction files populated by each reviewer were compared and disagreements were resolved by discussion and mutual consent.

### Datasets

After removing duplicate entries, we identified a list of resources, articles published based on the resources, bibliometric data for the articles and bibliometric data for researchers who published the articles.

1. Resource dataset

We reviewed the methods section of each article to extract information about each resource. We also reviewed and extracted information available on their individual websites (if present) and internet in general (using Google search). We categorized the data for each resource using the following categories:

1. Type of resource: Resources developed/initiated as a part of a research project/study were categorized under 'Study specific’, those developed/initiated by a department in an organization were categorized under 'Departmental’, those developed through an institutional/organizational initiative or national initiative were categorized under 'Institutional’ and national initiative respectively. We evaluated
2. Type of affiliation: We analyzed the location of each resource and categorized its institutional affiliation into six sub-categories based on presence in Government owned academic institutes/hospitals (Example: A Government owned Medical College and attached Tertiary Care Municipal hospital), Privately owned and managed Academic Institute/hospitals (Example: A Private Medical College and attached Tertiary Care Hospital), Privately owned and managed non - Academic Institute/hospitals (Example: A Privately owned Tertiary Care Hospital), For profit private organizations (Example: a resource owned by a Pharma company), Government organizations (Example: A resource owned by the Health ministry) and Non Government organization/Society/Associations (Example: A resource owned by a national cardiology society)
3. Location state: We analyzed the geographical location of each resource and categorized it as per states and zones. For the purpose of facilitating analysis, we divided India into four zones viz: North, East, West and South.
4. Type of disease: By analyzing the articles retrieved and reviewing any additional information available on the web, we identified the disease type for which data was collected in the resource
5. Total number of articles published based on data of each resource

2. Bibliometric dataset

For each individual resource, we identified the total number of articles published till date (Nov 18, 2012). Next for each article, we extracted data on journal name, corresponding journal impact factor and citations received. Journal name was identified from the full citation of the article. We extracted data on journal impact factor from each individual journal website or referred to the ISI Thomson impact factor database ((Thomson Reuters | The Thomson Reuters Impact Factor | Science 2013). We extracted data on the total citations received by each article till date (Nov 18, 2012) by referring to Google scholar.

3. Researcher dataset

From the 133 articles, we extracted the names of first and last authors, their institutional affiliations, location details (city, state, country) and email addresses.

Finally we carried out descriptive analysis of the 3 datasets described above.

## Results

### Search results

**Figure 1:**
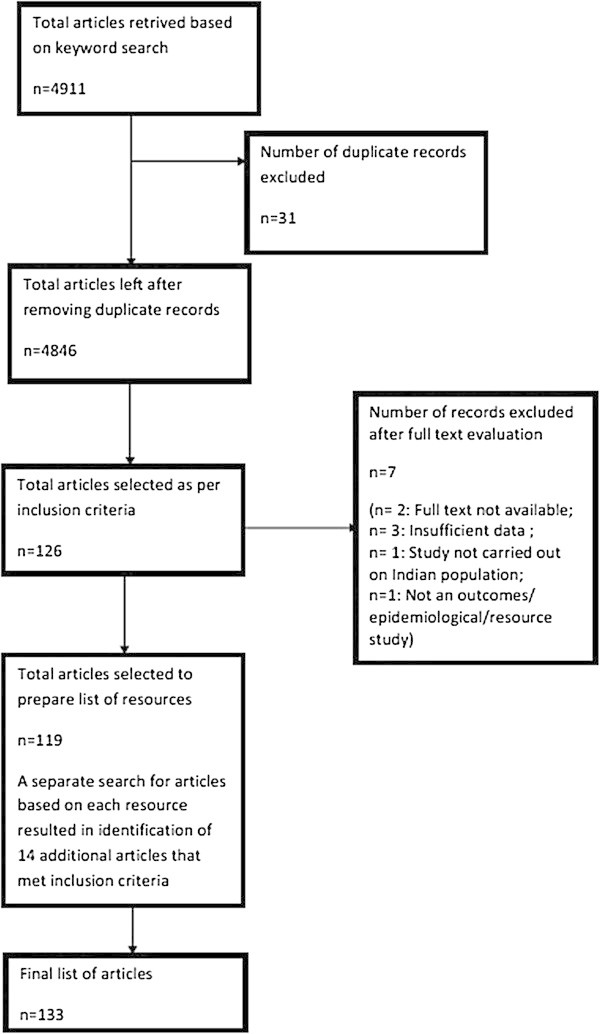
**Flowchart describing review and article retrieval process.**

We identified a total of 4911 articles based on keyword search. After removing duplicate entries (31) we were left with 4846 articles for review. After reviewing titles and abstracts of articles and applying selection criteria, we shortlisted 126 articles. Next, we reviewed the full text of 126 articles and excluded 7 articles dissatisfying our selection criteria thus yielding a total of 119 articles which were selected for inclusion in the study. After analyzing these 119 articles, we were able to identify a total of 91 unique resources. 14 more articles were retrieved through a secondary search carried out with an aim of identifying additional publications related to the resources reported in the articles. This increased the tally to 133 articles. We were able to identify a total of 91 unique resources and 193 researchers who had published outcomes research articles using these resources. (Figure 1: Flowchart describing review and article retrieval process)

- Resource data
- Type of resource
  Our analysis reveals that most of the resources are registries (62/91) and databases (23/91) [Table 1].
- Location
  Analysis of geographical distribution of the resources reveal that most of them are located in Maharashtra (19/91), Tamil Nadu (11/91), Chandigarh(8/91) and Kerala(7/91) States of India. Analysis on zonal perspective revealed their predominant presence in Southern (32/91) and Western (26/91) zones [Table 2].
- Initiative driving/supporting the resource
  Analysis of the affiliation data for each resource reveal that most of the resources are either institutional (38/91) or regional (35/91) initiatives [Table 3]. Further, analysis of their affiliation data also revealed that they were present in Government owned academic institutes/hospitals (57/91) and privately owned and managed Non - Academic Institute/hospitals (14/91) [Table 4].
- Type of disease
  Analysis of data collected by each resource and publications based on them reveal that most of the resources are collecting data on Cancer (44/91) followed by Stroke (5/91) and Diabetes (4/91) [Table 5].
- Bibliometric data
- Journal analysis
  Our analysis revealed that most of the publications based on data from the 91 resources were published in international journals (139/193).
- Citation analysis
  We observed that the publications based on these resources received an average of 0–22 citations (55/91) and 21–40 citations (17/91) [Table 6].
- Journal impact factor analysis
  We noted that the articles using the data from the 91 resources were usually published in journals with an impact factor of 0–1.99 (43/91) and 2–3.99 (18/91) [Table 7].
- Researcher data
- Location of outcomes researchers
  Analysis of geographical location data for each researcher revealed that they are primarily located in Maharashtra (37/193), Tamil Nadu (24/193), Chandigarh (16/193) and Karnataka (16/193) States. They are predominantly located in southern (76/193) and western zones (47/193) of India. Some of the authors (17/193) are located outside India [Table 8].
- Affiliation of outcomes researchers
  Analysis of the affiliation data for the outcomes researchers reveal that more than half of them are working in Government owned and managed Academic Institutes/Hospitals (96/193) and some in Privately owned and managed Academic Institutes/Hospitals (35/193). Some of them were affiliated to organizations outside India (10/193) [Table 9].

**Table 1:** **Type of resources**

Category of resources	Number of resources
Registry	62
Database	23
Medical records	3
Electronic medical records	2
Database, hospital information system	1

**Table 2:** **Geographical distribution of resources by zones and states**

Zone	States	Number of resources
West	Maharashtra	20
South	Tamil Nadu	11
North	Chandigarh	8
South	Kerala	8
North	Delhi	7
South	Karnataka	7
South	Andhra Pradesh	5
West	Gujarat	5
North	Uttar Pradesh	5
East	Assam	4
East	West Bengal	3
South	Pondicherry	2
West	Chattisgarh	1
West	Madhya Pradesh	1
East	Manipur	1
East	Mizoram	1
Not Available	Not Available	1
North	Rajasthan	1
East	Sikkim	1

**Table 3:** **Initiatives driving the resources**

Type of resources	No. of resources cited
Institutional	38
Regional	35
Study specific	13
National	3
Not available	2

**Table 4:** **Affiliation of resources**

Institutional type based distribution of resource publications	Number of researchers
Government owned and managed Academic Institute/hospital	57
Privately owned and managed non - Academic Institute/hospital	14
Privately owned and managed Academic Institute/hospital	8
Non Government organization/Society/Association	5
Government organization	3
Government owned and managed Academic Institute	2
Not available	2

**Table 5:** **Resources by type of diseases**

Disease	No. of resources cited
Cancer	44
Stroke	5
Diabetes	4
Not Applicable	4
Acute Coronary Syndrome	3
Hypertension	3
Corneal ulcer	2
Haemophilia	2
Diseases with one resource each (Adult respiratory distress syndrome/Acute Lung Injury, AIDS, Burns, Chronic Kidney Disease, Chronic Heart Disease, Coronary restenosis, Deafness, Delirium, Dementia, Distal-type Cervical Spondylotic Amyotrophy, Endopthalmitis, Epilepsy, Gallstone, Hemolytic Uremic Syndrome, Kawasaki Disease, Kidney diseases, Leprosy, Mental Disorder, Myocardial Infarction, Necrotizing Pancreatitis, Obesity, Rheumatic disease, Vitiligo, Zygomycosis)	1

**Table 6:** **Citations received by resource based publications**

Total citation group	No. of resources cited
0 – 20	55
21 – 40	17
41 – 60	4
61 – 80	2
81 – 100	3
101 – 120	4
121 – 140	1
160 – 180	1
180 – 200	1
Above 200	3

**Table 7:** **Journal impact factor analysis of articles published based on data derived from resources**

Sum JIF1 group	No. of resources cited
0 - 1.99	43
2 - 3.99	18
4 - 5.99	6
6 - 7.99	10
8 - 9.99	3
10 - 11.99	1
12 - 13.99	2
14 - 15.99	1
16 - 17.99	1
18 - 19.99	1
Above 20	2
Above 30	2
Above 40	1

**Table 8:** **Geographical distribution of outcomes researchers in India – by zone into state**

Zone	States	Count of unique authors
West	Maharashtra	37
South	Tamil Nadu	24
South	Kerala	17
North	Chandigarh	16
South	Karnataka	16
South	Andhra Pradesh	13
North	Delhi	13
North	Uttar Pradesh	12
NA	NA	9
NA	Out of India	9
East	West Bengal	8
West	Gujarat	6
South	Pondicherry	6
West	Chattisgarh	2
West	Madhya Pradesh	2
North	Rajasthan	2
East	Sikkim	1

**Table 9:** **Affiliation of outcomes researchers**

Affiliation type	Count of unique authors
Government organization	1
Government organizationGovernment organization	1
Government owned and managed Academic Institute/hospital	96
NA	10
Non Government organization/Society/Association	20
Privately owned and managed Academic Institute/hospital	35
Privately owned and managed non - Academic Institute/hospital	30

## Discussion

To the best of our knowledge, this is the first study that carried out a systematic analysis of outcomes research resources in India as reported in published literature. We collected information relevant to 91 outcomes research resources in India and report details about each resource, bibliometric data of publications derived from these resources and researchers that conducted research studies using data derived from these resources.

We observed a predominance of registries and databases in India. Research registries collect long term clinical, health services and epidemiological data for a given population. They are essential to understand clinical and epidemiological trends as well as useful for policy analyses, planning and management of health care resources. (Roos and Nicole, Roos and Nicol 1999) (Broemeling et al. 2009). Databases are usually study specific or project specific. They are usually designed to collect data to answer a specific research question. The low number of EMR, EHR and HIS in India might be because of the fact that India has been slow in the adoption of biomedical and research informatics tools. Although having a wide range of advantages (Fraser et al., 2005) (Lobach and Detmer, 2007) (Mildon and Cohen, 2001) (Rustagi and Singh, 2012) concerns about privacy, reduction in clinical productivity, being resource intensive, (Rustagi and Singh, 2012) (Kluger, 2009) high purchase and maintenance costs make their adoption slower (Jha et al., 2009) (Hillestad et al., 2005).

We observed a geographical predominance of resources and researchers in southern and western zones indicating an imbalance. This imbalance may be further complicated by the fact that researchers from one zone may not have access to data from a resource located in another zone. This may significantly influence policy and funding decisions further resulting in a vicious cycle of resource duplication, under utilization of resources, and wastage of funding.

We also observed a predominance of Institutional and regional initiatives in spearheading/managing the resources. Although this trend is noteworthy and beneficial, it reflects small scale and medium scale research projects. National registries have their own importance in nationwide policy decisions as data cannot always be extrapolated from regional data. There are numerous examples of large scale nationwide initiatives like Nationwide inpatient sample (HCUP-US NIS, 2013), National Health Insurance Research Database (NHRI, Taiwan, 2013), Disease registries maintained by National Registry of Diseases Office (NRDO, Singapore, 2013) that have and continue to significantly contribute to national healthcare decision making and planning as well as in the improvement of quality of healthcare. Thus a balanced distribution of regional and national resources is essential. We also noted that most of the resources and researchers were located in Government or Privately owned academic organizations. Although a good trend, these organizations usually serve the urban population and provide tertiary care. Given the fact that India is largely an agrarian country, equitable distribution of resources into urban and rural areas would facilitate the collection of data that is truly representative of the Indian population. Policies derived from such a representative sample will be more effective than those based on extrapolated data that do not represent real life scenarios.

Most of the resources collected data on Cancer, Stroke and Diabetes. Given the significant rise in cancer, cardiovascular and metabolic disorders in India, (Takiar et al., 2010) (Young et al., 2009) this distribution appears to be moving in the right direction. Yet, it should be noted that there exists a vast difference amongst number of resources in each of these groups indicating a predominance of cancer resources. Accordingly, it prepares the case for the need of similar outcomes data resources for nationally prevalent diseases like Malaria, Tuberculosis. This can be implemented by incorporating relevant outcomes data variables in surveillance and national programs.

A predominance of publications derived from the short listed resources in international journals is a good trend as it helps disseminate results to a global audience. Yet the Journal impact factor (JIF) and citation index of these publications may be indicative of the quality and impact of results published. Training programs to help clinicians and researchers collect data using global accepted data standards and report them using standard reporting guidelines may make future publications reach a larger audience and gain higher impact. In this regard, a workshop on imparting outcomes research skills to medical faculty members was recently conducted with the aid of Indian Council of Medical Research. (Savardekar L, Shah J, Bacchav S, Kshirsagar N, Translating Ideas into Research Projects and Manuscripts in Outcomes Research:Experiences of An ICMR Workshop. unpublished observations).

Most of the registries and databases identified through this study have not been explored to their true potential. In most cases, data from these registries have resulted in one to three publications. Further, most of them do not have their own websites or web pages within their organization. Sharing of data dictionaries or actual data – a norm of current times is hardly applicable to these resources. This demonstrates that detailed information about these resources is not easily accessible. The Department of Science and Technology (India) conducted a National Survey on Resources Devoted to Science &Technology Activities (National Science & Technology Management Information System, India, 2013) but the resultant data is not publicly available. Secondly, the survey questionnaire does not capture granular information about research resources. Finally, we are not aware about its utility and effectiveness in facilitating collaborations and guiding policy decisions at a state and national level. It is thus evident that awareness of existing outcomes research resources in India is low thus impairing the ability of 1. Researchers and research groups to optimally utilize existing outcomes data for carrying out outcomes research studies and 2. Research policy makers to utilize resource availability and resource performance data while making resource allocation decisions.

### Limitations

All efforts were made to do an exhaustive review of the literature but given the nature of research question and limitations in terms of keywords and filters, we may have missed relevant publications reporting information about outcomes research resources. Secondly, data resources and researchers are not the only factors that contribute to outcomes research. Factors like skilled manpower, training opportunities, availability of funding, institutional policy have a role to play. Since this information is not readily available on the web or in publications, we interpreted based on the data that was available to us. There is a need for national level initiatives to collect data about the location, capabilities and performance of outcomes research resources. Thirdly, we did not include keywords related to surveillance data in our search strategy as surveillance in itself is a huge area and beyond the scope of this project. We intend to pursue this in a subsequent study. Finally, although semi automated methods like natural language processing and computational ontologies could have been utilized to carry out data extraction and reasoning of data extracted from published articles (Lin et al., 2010) (Ceci et al., 2012), we preferred the manual method as 1. The number of relevant articles identified through an initial review was low and 2. To ensure higher quality of data abstraction.

## Conclusion

Given the importance of Outcomes research, relevant resources should be supported and encouraged which would help in the generation of important healthcare data that can guide health and research policy. Clarity about the distribution of outcomes research resources can facilitate future resource and funding allocation decisions for policy makers as well as help them measure research performance over time.

## Electronic supplementary material

Additional file 1: **Outcomes research resources identified.** (DOC 73 KB)

## Abbreviations

ACS: Acute coronary syndrome
MI: Myocardial infarction
Ob-Gyn: Obstetrics & gynecology
HIV AIDS: Human immunodeficiency virus infection/Acquired immunodeficiency syndrome
NA: Not applicable
EMR: Electronic medical records
EHR: Electronic healthcare records
HIS: Health information system.
