# Supplementary material for: Outcomes research resources in India: current status, need and way forward
Source: Springerplus. 2013 Oct 7;2:518. doi: 10.1186/2193-1801-2-518 (PMC3804670; doi:10.1186/2193-1801-2-518)
Supplement: Supplementary file 1 — Additional file 1: Outcomes research resources identified. (DOC 73 KB) [file 40064_2013_588_MOESM1_ESM.doc]

**Appendix 1.** Outcomes research resources identified

| Acute Stroke Registry, Jawaharlal Institute of Post Graduate Medical Education and Research, Pondicherry |
| --- |
| Asian Indian Donor Marrow Registry, Delhi |
| Bivaflo Registry, Delhi |
| Cancer Registry, S.M.S. Medical College, Jaipur |
| Case records, Regional Leprosy Training & Research Institute (RLTRI), Raipur |
| Clinical database of Kidney diseases, Post Graduate Institute of Medical Education & Research (PGIMER), Chandigarh |
| Clinical records, Haemophilia clinics at Mumbai, Pune and Kolhapur |
| Computerized database, Aravind Eye Hospital and Post Graduate Institute of Ophthalmology, Pondicherry |
| Cornea transplants database, Post Graduate Institute of Medical Education and Research, Chandigarh |
| Corneal ulcer database, LV Prasad Eye Institute, Hyderabad |
| COSTAR I Trial Registry, Delhi |
| CREATE registry, Bangalore |
| Database of patients with Squamous cell carcinoma of the gingivo-buccal complex, Tata Memorial Hospital, Mumbai |
| Database, Emergency Management and Research Institute (EMRI), Ahmedabad |
| Datasets of the Department of Health and Family Welfare, Government of Gujarat (GOG), Gandhinagar |
| Deliberate Self harm register, Institute of Psychiatry, Kolkata |
| Dementia registry, Nizam's Institute of Medical Sciences, Hyderabad |
| Department of Pathology Registry, Moti Lal Nehru Medical College, Allahabad |
| Departmental database, Department of Surgical Gastroenterology, Sanjay Gandhi Postgraduate Institute of Medical Sciences |
| Diabetes Electronic Medical Records, Dr. Mohan's Diabetes Specialities Centre (DMDSC), Chennai |
| Diabetes registry, Diabetes Research Center, Chennai |
| Dindigul Ambilikkai Cancer Registry, Chennai |
| Elaxim Indian Registry, Bangalore |
| Electronic database, Regional Cancer Centre, Trivandrum |
| Electronic Head and Neck Cancer Database, Tata Memorial Hospital, Mumbai |
| Electronic Medical And Hospital Records, Tata Memorial Hospital, Mumbai. |
| Endophthalmitis Registry, L. V. Prasad Eye Institute, Visakhapatnam |
| Hemophilia database, University of Pune, Pune |
| High Risk Register, All Yavar Jung National Institute for the Hearing Handicapped, Mumbai |
| Histopathology Department Registry, Bombay Hospital and Medical Research Centre, Mumbai |
| Histopathology Reporting Registry, Tamil Nadu Government Dental College and Hospital, Chennai |
| Hospital Based Cancer Registry, Bangalore |
| Hospital Based Cancer Registry, Cancer Institute (WIA), Chennai |
| Hospital Based Cancer Registry, Dibrugarh District |
| Hospital Based Cancer Registry, Mumbai |
| Hospital Based Cancer Registry, Trivandrum |
| Hospital Based Stroke Registry, National Neurosciences Centre, Kolkata |
| Hospital Cancer Registry, Rajiv Gandhi Cancer Institute and Research Center, Delhi |
| Hospital Cancer Registry, Tata Memorial Hospital, Mumbai |
| Hospital Registry, CARE Hospitals, Hyderabad |
| Indian Chronic Kidney Disease (CKD) Registry, Nadiad |
| Indian Transplant Registry, Indian Society of Organ Transplantation, Chennai |
| Institute Transplant Registry, Dr H.L. Trivedi Institute of Transplantation Sciences (ITS), Ahmedabad |
| Institutional Database of Craniotomies, Tata Memorial Centre, Mumbai |
| Institutional Database, Department of Neurological Sciences, Christian Medical College, Vellore |
| Institutional Database, Sanjay Gandhi Postgraduate Institute of Medical Sciences, Bangalore |
| Institutional Zygomycosis Registry, Postgraduate Institute of Medical Education & Research, Chandigarh |
| Karnataka Diabetes Registry, Bangalore |
| Kawasaki Disease Registry, Indian Academy of Paediatrics, Mumbai |
| Kerala ACS Registry, Kerala Chapter, Cardiological Society of India |
| Kerala Registry of Epilepsy and Pregnancy, Sree Chitra Tirunal Institute of Medical Sciences and Technology, Trivandrum |
| Leukaemia-Lymphoma Registry, K. G. Medical College, Lucknow |
| Medical Records, Dr. B. R. A. Institute Rotary Cancer Hospital, Delhi |
| Multicenter Registry of High-Risk Percutaneous Coronary Intervention and Adequate Platelet Inhibition |
| Mumbai Stroke registry |
| Natural Background Radiation Cancer Registry Karunagappally (Karunagappally Rural Cancer Registry ) |
| Pancreaticoduodenectomy Database, Lilavati Hospital and Research Centre, Mumbai |
| Pathology database and Hospital Information System, Sanjay Gandhi Post Graduate Institute of Medical Sciences, Lucknow |
| Patient database, Postgraduate Institute of Medical Education and Research, Chandigarh |
| Patient Records Database, Tata Memorial Hospital (TMH), Mumbai |
| Pigmentary Clinic Database, Postgraduate Institute of Medical Education and Research, Chandigarh |
| Population Based Cancer Registry (PBCR) Ahmedabad Urban agglomeration area |
| Population Based Cancer Registry (PBCR), Aurangabad |
| Population Based Cancer Registry (PBCR), Bangalore |
| Population Based Cancer Registry (PBCR), Bhopal |
| Population Based Cancer Registry (PBCR), Cachar District (Silchar Cancer Registry) |
| Population Based Cancer Registry (PBCR), Chennai |
| Population Based Cancer Registry (PBCR), Delhi |
| Population Based Cancer Registry (PBCR), Dibrugarh District |
| Population Based Cancer Registry (PBCR), Kamrup Urban District |
| Population Based Cancer Registry (PBCR), Kolkata |
| Population Based Cancer Registry (PBCR), Manipur State (Imphal West District Registry) |
| Population Based Cancer Registry (PBCR), Mizoram State |
| Population Based Cancer Registry (PBCR), Mumbai |
| Population Based Cancer Registry (PBCR), Nagpur |
| Population Based Cancer Registry (PBCR), Pune |
| Population Based Cancer Registry (PBCR), Sikkim State |
| Population Based Cancer Registry (PBCR), Trivandrum |
| Population-based Hereditary Cancer Registry, Cancer Institute (Women’s India Association), Chennai |
| Prospective database at Minimal Access, Metabolic and Bariatric Surgery Centre, Sir Ganga Ram Hospital, Delhi |
| Prospective database in Respiratory ICU, Postgraduate Institute of Medical Education and Research, Chandigarh |
| Prospective database, at Department of Surgical Gastroenterology, Sanjay Gandhi Postgraduate Institute of Medical Sciences |
| Prospective database, Institute of Surgical Gastroenterology and Liver Transplantation, Government Stanley Medical College Hospital, Chennai |
| Referral register, Postgraduate Institute of Medical Education and Research, Chandigarh |
| Registry of Burn patients, Wenlock District Hospital, Mangalore |
| Rheumatic Fever and Rheumatic Heart Disease Registry, Postgraduate Institute of Medical Education and Research, Chandigarh |
| Rural cancer registry, Barshi |
| STEMI - Indian Registry |
| Trivandrum Stroke Registry, Sree Chitra Tirunal Institute for Medical Sciences, Trivandrum |
| Venous Stroke Registry, Nizam's institute of medical sciences, Hyderabad |
| YRG CARE Chennai HIV Observational Database (YCHOD) |
